# Small Bowel Adenocarcinoma Complicating Coeliac Disease: A Report of Three Cases and the Literature Review

**DOI:** 10.1155/2012/935183

**Published:** 2012-12-01

**Authors:** Hafida Benhammane, Fatima Zahra El M'rabet, Karima Idrissi Serhouchni, Mounia El yousfi, Ilias Charif, Imane Toughray, Naoufal Mellas, Afaf Riffi Amarti, khalid Maazaz, Sidi Adil Ibrahimi, Omar El mesbahi

**Affiliations:** ^1^Department of Medical Oncology, Hassan II University Hospital, Fez, Morocco; ^2^Department of Pathology, Hassan II University Hospital, Fez, Morocco; ^3^Department of Hepato-Gastroenterology, Hassan II University Hospital, Fez, Morocco; ^4^Department of General Surgery, Hassan II University Hospital, Fez, Morocco

## Abstract

Coeliac disease is associated with an increased risk of malignancy, not only of intestinal lymphoma but also of small intestinal adenocarcinoma which is 82 times more common in patients with celiac disease than in the normal population. We report three additional cases of a small bowel adenocarcinoma in the setting of coeliac disease in order to underline the epidemiological features, clinicopathological findings, and therapeutic approaches of this entity based on a review of the literature. The three patients underwent a surgical treatment followed by adjuvant chemotherapy based on capecitabine/oxaliplatin regimen, and they have well recovered.

## 1. Introduction

Malignant tumors of the small bowel fall in the category of rare neoplasm comprising only 3% of all gastrointestinal malignancies. Primary adenocarcinoma is the most common histological subtype constituting 35–50% of cases [1, 2]. Identified risk factors of small bowel adenocarcinoma (SBA) include Crohn's disease, coeliac disease, and genetic syndromes such as familial adenomatous polyposis, hereditary nonpolyposis colon cancer (HNPCC), and Peutz-Jeghers syndrome [3]. The association between coeliac disease (CD) and malignancies is well established with the reported frequency of malignancies ranging from 5% to 21%. SBA in association with CD was first reported in 1958 [4]. Etiology and pathogenic mechanisms have not been well elucidated.

In this paper, we describe our experience with three cases of SBA in the setting of CD with a brief review of the literature. 

## 2. Case Reports

**Case No. 1**

**Figure 1:**
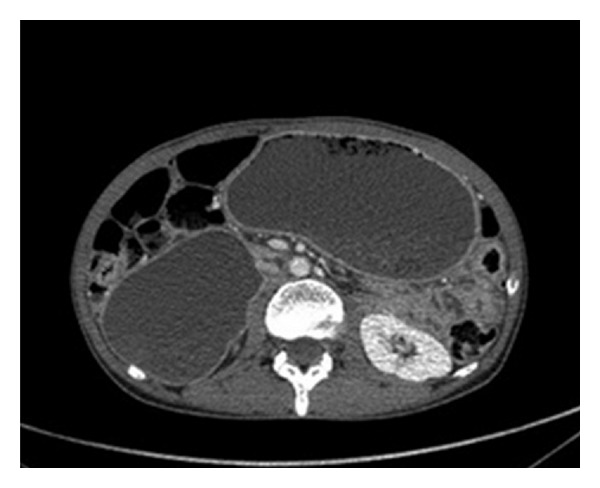
Abdominal computed tomography showing gastric and duodenal distension related to an irregular thickening with stenosis localized to genus inferius duodenal portion.

**Figure 2:**
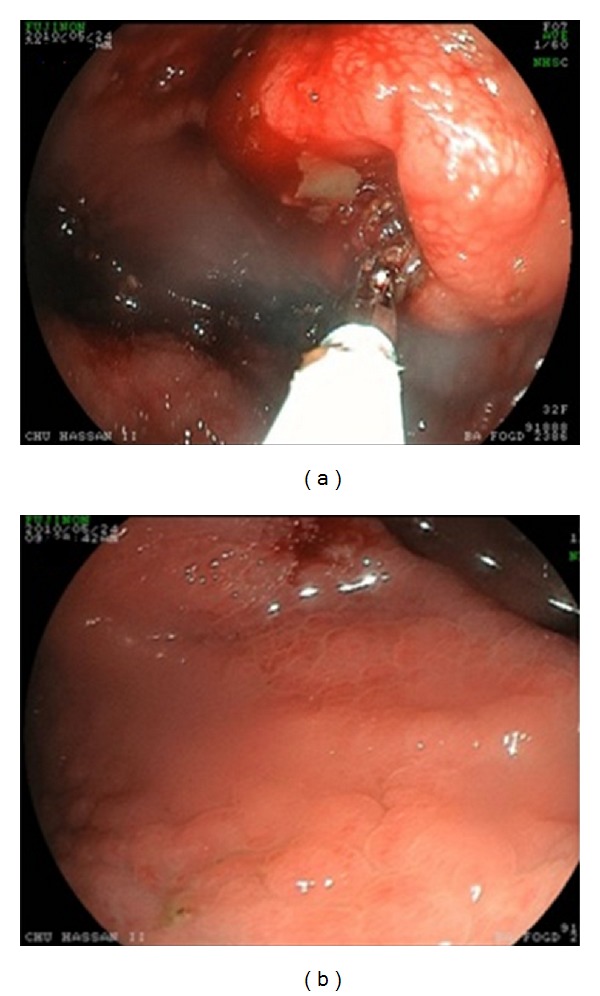
Upper gastrointestinal endoscopy revealing (a) duodenal stenosis with proximal dilatation, (b) mosaic mucosal pattern with scalloped configuration of duodenal folds.

**Figure 3:**
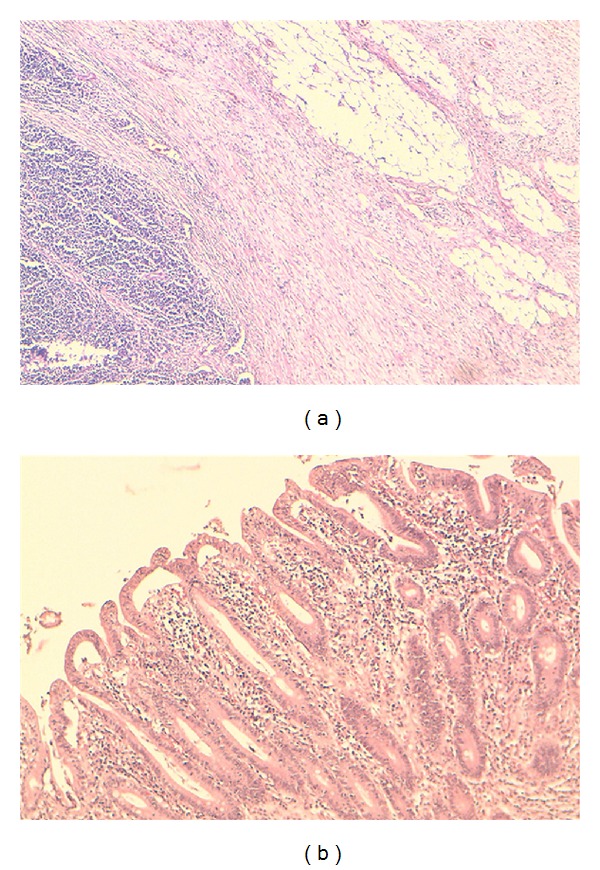
Histology of surgical specimen revealing (a) moderately differentiated adenocarcinoma of duodenum, (b) total villous atrophy, crypt hyperplasia, and inflammatory cells in adjacent mucosa—Marsch IV.

A. B., a 43-year-old woman had consulted for a 3-month history of epigastric pain and 10 Kg weight loss. Her past medical history included CD diagnosed one year earlier with strict adherence to the gluten-free diet (GFD). Abdominal computed tomography (CT) revealedan irregular duodenal thickening localized to genu inferius portion with proximal stenosis (Figure 1). Upper endoscopy showed a mosaic mucosal pattern and a scalloped configuration of duodenal folds associated to duodenal stenosis (Figure 2); biopsies identified duodenal adenocarcinoma. The patient underwent laparotomy which revealed a soft mass of duodenal genu inferius. Neither metastatic lesions of the liver nor peritoneal implant was evident. Cephalic pancreaticoduodenectomy with regional lymphadenectomy was successfully performed. Histological analysis of the specimen resection confirmed the diagnosis of moderately differentiated duodenal adenocarcinoma with serosal invasion; resection margins and lymph nodes were free of tumor; the adjacent duodenal mucosa showed subtotal villous atrophy (Figure 3). The tumor was classified as pT3 N0 M0. Regarding the young age of the patient and the duodenal localization of the tumor, adjuvant chemotherapy based on capecitabine/oxaliplatin (CAPOX) regimen was prescribed. Currently, the patient is alive without any evidence of recurrence, 20 months after the operation.

**Case No. 2**

M. A., a 46-year-old man had consulted for abdominal pain and weight loss for 6 months duration. Physical examination revealed a 7 × 6 cm sized abdominal mass arising from the left iliac fossa. Abdominal CT demonstrated intestinal tumoral thickening of the left iliac fossa and mesenteric lymph nodes. Upper fibroscopy and colonoscopy were normal. At upper enteroscopy, we found endoscopic appearance suggestive of CD. Biopsies confirmed the diagnosis. The patient underwent laparotomy which revealed the presence of a locally advanced tumor of the ileum involving recto-sigmoid junction with lymphadenopathy without peritoneal implant or liver metastasis. Small bowel resection and sigmoid resection with terminoterminal anastomosis were performed. The diagnosis of poorly differentiated adenocarcinoma of the ileum was retained following immunohistochemical analysis of resected specimen; so the tumor was classified as pT4 N1 M0. Given the lymph node metastasis, adjuvant chemotherapy based on CAPOX regimen was received. Currently, the patient remains well on GFD.

**Case No. 3**

B. K., A 37-years-old man was admitted to the emergency room with acute intestinal obstruction. Questioning revealed two months history of vomiting, diarrhea, and abdominal pain. Abdominal CT showed stenosis of the last ileal loop with proximal dilatation. The patient underwent an emergent laparotomy which revealed an obstructive tumor of the ileum; a segmental resection with anastomosis and regional lymphadenectomy were performed. The histological diagnosis was a moderately differentiated adenocarcinoma of the ileum classified as pT2 N1 M0. Diagnosis of CD was made retrospectively 1 month later at upper endoscopy and was confirmed by biopsy and serological tests, and then GFD was started. Adjuvant chemotherapy based on CAPOX regimen was received. Currently, the patient responds to institution of GFD, and he remains well 56 months after surgery.

## 3. Discussion

Small bowel malignant tumors are uncommon malignant neoplasms accounting for only 3% of all gastrointestinal malignancies [1]. However, its incidence appears to be increasing according to a recent analysis of the Surveillance Epidemiology and End Results (SEER) demonstrating an increasing overall incidence from 11.8 cases per million in 1973 to 22.7 cases per million in 2004 in the USA [2]. Primary adenocarcinoma is the most common histological subtype constituting 35–50% followed in decreasing order by carcinoid tumors (30%), lymphomas (15%), and gastrointestinal stromal tumors and other sarcomas (10%) [2, 5].

Small intestinal malignancies have been observed to be more common in people with a number of inflammatory bowel diseases and genetic syndrome. Crohn's disease, coeliac disease, Peutz-Jeghers syndrome, familial adenomatous polyposis, and HNPCC are known predisposing factors for SBA [3, 5]. Coeliac disease(CD) is an autoimmunediseasedue to gluten intolerance which is associated with an increased risk of SBA [6]. This association has been confirmed by a large collaborative study on 235 coeliac patients in the UK, and the relative risk for small bowel adenocarcinoma was 82.6 [5]. In addition, Howdle et al. through the analysis of 175 cases of SBA found an associated CD in 13% [7]. Currently, SBA is now known to be the second most common invasive malignancy after lymphoma in coeliac patients [6].

Although both etiology and pathogenetic mechanisms of SBA in coeliac setting remain unclear, the following explanations have been suggested: a high turnover of the inflammatory population with mucosal lymphocyte infiltration, an increased permeability to oncogenic factors, a malabsorption of protective substances such as vitamins A and E, or an impaired immune surveillance [3]; additionally, because of the histological similarities with adenocarcinoma arising in the colon, it has been suggested that adenocarcinoma of the small bowel in CD arises through an adenoma-carcinoma sequence [6]. However, despite some reported cases, this hypothesis remains controversial. 

Usually, SBA is most commonly located in the duodenum (55%), followed by the jejunum (30%) and the ileum (15%) [8]. However,; in coeliac patients these carcinomas tend to develop in the jejunum and are more likely to develop as an adenoma-carcinoma sequence than as dysplasia in flat mucosa [6].

Development of carcinoma is well recognized in association with long-standing gluten enteropathy; nevertheless, it can occur in patients with no history suggestive of a malabsorption syndrome, and the CD is diagnosed until the resection of SBA when the histological analysis of the specimen resection shows villous atrophy in adjacent nonneoplastic mucosa [9]. In our series, we had two cases in which the malignancy was the first presentation of CD.

Because of the inaccessibility of the small bowel to routine endoscopy, diagnosis of SBA is usually made at an advanced stage (74% stage III or IV) [3]. Symptoms are not specific and should be carefully checked in coeliac patients; they include anemia which is the most common presenting feature, abdominal pain, weight loss, gastrointestinal bleeding, or vomiting. In some cases, the tumor is revealed by a complication such as an occlusion or a perforation [8]. Because an early diagnosis is crucial for curative surgery, coeliac patients suspected of having SB neoplasm must be evaluated by endoscopic and/or radiological techniques. Endoscopic techniques with biopsy are standard methods for diagnosis of SB tumors; upper endoscopy can detect lesions of duodenum and proximal jejunum, and colonoscopy can examine the terminal ileum. However, endoscopy is limited by the nonviewing of the entire SB [3]. Recently, capsule video endoscopy (CVE) has become an important tool in the investigation of patients with small bowel diseases. In a series of 47 coeliac patients with a high risk of complication, CVE has detected lesions in 45% of cases including one adenocarcinoma. In addition, this technique has the great advantage of being a noninvasive technique and to visualize the entire small bowel [10]. Furthermore, small intestinal barium and double-contrast study findings are not pathognomonic and can be difficult to interpret in the context of CD with an accuracy of 30–44%; other radiological investigations (CT, MRI, and endoscopic sonography) have a major interest in staging [3].

No consensus on treatment has been yet documented; the only available treatment of SBA is surgery with an overall rate of curative resection of 40–65% [11]. Because of its rarity, very little data has been published regarding the value of chemotherapy and radiotherapy in the adjuvant or advanced setting. In fact, current therapeutic options are based on an extrapolation from data observed in colon cancer [2, 4, 11].

Does the gluten-free diet (GFD) protect against recurrence? There is evident data suggesting that GFD has a significant role in reducing risk of CD-related gastrointestinal malignancies, but the protective role of GFD against recurrence in patient previously treated for SB neoplasia is controversial, and there are three reports of CD patients who developed a second metachronous SBA 15, 9, and 2 years after a presentation with a first SBA despite a strict adherence to GFD [12, 13]. These data suggest that small bowel surveillance in celiac patients with a history of SBA may be useful, but this will require further study.

## 4. Conclusion

These cases confirm that CD is associated with a definite increase in the risk of developing SBA, which represents an entity with specific characteristics. No consensus on treatment is available. As the prognosis is not uniformly poor and some patients are potentially curable by total resection, as in our patients, there is evidence that an early diagnosis is crucial to improve the outcomes of this malignancy; but at present, there are no recommendations for screening in CD patients. In the light of this literature and as clinicians, we must pay attention to CD patients with vague symptoms and bear in mind that this could be related to malignant complication.

## Abbreviations

SB: Small bowel
SBA: Small bowel adenocarcinoma
CD: Celiac disease
HNPCC: Hereditary nonpolyposis colon cancer
GFD: Gluten-free diet
CT: Computed tomography
CAPOX: Capecitabine/oxaliplatin
CVE: Capsule video endoscopy.
